# Supplementary material for: Daily vocal exercise is necessary for peak performance singing in a songbird
Source: Nat Commun. 2023 Dec 12;14:7787. doi: 10.1038/s41467-023-43592-6 (PMC10716414; doi:10.1038/s41467-023-43592-6)
Supplement: Supplementary file 1 — Supplementary Information [file 41467_2023_43592_MOESM1_ESM.pdf]

Supplementary Information for

Daily vocal exercise is necessary for peak performance singing in a songbird

Iris Adam[1,*], Katharina Riebel[2], Per Stål[3], Neil Wood[4], Michael J. Previs[4], Coen P.H. Elemans[1,*]

Affiliations:

[1]Department of Biology, University of Southern Denmark, Denmark.

[2]Institute of Biology, Animal Sciences & Health, Leiden University, The Netherlands.

[3]Department of Integrative Medical Biology, Umea University, Sweden.

[4]Department of Molecular Physiology and Biophysics, Larner College of Medicine, University of Vermont, Burlington, USA.

*Corresponding authors. Email: irisadam@biology.sdu.dk; coen@biology.sdu.dk

This PDF file includes:

1 Supplementary Text

1.1 Fibre types in syringeal muscles

1.2 Syringeal muscle proteome

2 Supplementary Figures

2.1 Supplementary Figure 1

2.2 Supplementary Figure 2

2.3 Supplementary Figure 3

3 Supplementary Tables

3.1 Supplementary Table 1

3.2 Supplementary Table 2

4 Supplementary References

Supplementary data for this manuscript include the following separate xlsx files:

Supplementary Data 1: Summary of statistical tests

Supplementary Data 2: Proteomics: LC peaks of intact and denervated DTB muscle. LC peaks of DTB muscle after 7 days of singing prevention.

1 Supplementary Text

1.1 Fibre types in syringeal muscles

Songbird syringeal muscles contain two fibre types; fast and superfast[1,2], but is currently not known if, and how these two syrinx muscle fibre types respond to exercise paradigms. In zebra finch males the majority (67–87%) of all muscle fibres is classified as superfast muscle (SFM) fibres[1,2] and not immunoreactive to any available antibodies raised against heavy myosin chain (MyHC) isoforms. The remaining 13–33% of syringeal muscle fibres are smaller diameter fibres immunoreactive to an antibody recognizing mammalian fast twitch MyHCs (MY-32)[1,2].

1.2 Syringeal Muscle Proteome

To identify and quantify disuse-induced protein expression changes, we performed proteomic profiling using liquid chromatography mass spectrometry in unmanipulated syringeal muscles (see Methods). We categorized the identified proteins according to their role in 1) force production (sarcomeric), 2) calcium handling and 3) mitochondrial function. We identified 450 proteins with high confidence (**Data S2**) of which over 80% were sarcomeric, calcium handling and mitochondrial proteins (**Fig 2ef**).

As part of the sarcomeric proteins we detected five MyHC isoforms: MYH7 (slow), MYH13 (superfast) and 3 other MyHCs encoded by genes in the fast cluster (Uniprot accessions A0A674GPZ1, A0A674GUX9, A0A674H378). With 92±3% of the total myosin pool, MYH13 was the most abundant MyHC, conforming earlier RNA-based observations[1], and the highest MYH13 fraction found in any muscle to date. Myosin light chains 1 and 2 (MYL1, MYL2, **Fig 2hi**) and troponin subunits TNNC2, TNNI2 and TNNT3 resembled typical mammalian fast twitch striated muscle. Furthermore, calcium handling proteins involved in calcium release and reuptake (SERCA1 and RYR1) were also identical to mammalian fast twitch isoforms. Interestingly, parvalbumins - cytosolic calcium buffers - were expressed an order of magnitude higher than in mouse limb muscle[3] **(Fig 2j)**. This provides a mechanism allowing fast muscle relaxation during bouts of muscle activity in song by pumping back temporarily sequestered cytosolic calcium back into the sarcoplasmic reticulum later between bouts of activity[4]. Mitochondrial proteins, such as proteins constituting ADP/ATP transporter (SLC25A4) and ATP-Synthase (ATP5F1A and B), were highly abundant (**Fig 2k**), making up 38±6% of the total proteome. Taken together, the high expression of MYH13, parvalbumins and mitochondrial proteins supports the superfast contraction speed and calcium transients as well as high energy demand of syringeal muscles.

3 Supplementary Figures

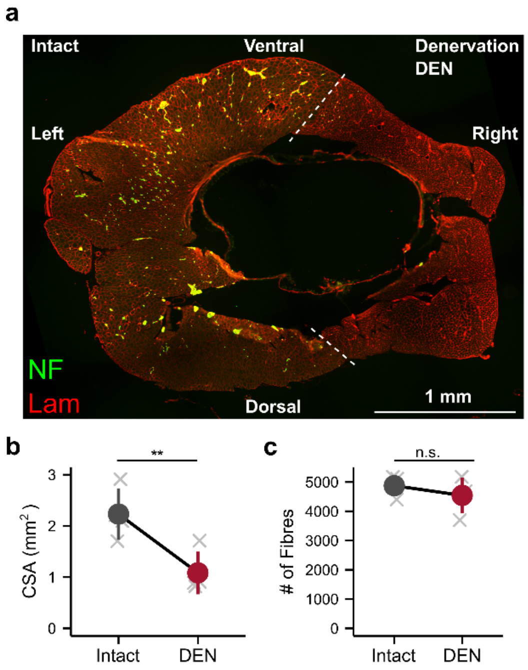

Supplementary Figure 1. Denervation effects on syrinx cross-sectional area and muscle fibre number. a, Immunohistochemical staining of a cross-section of the adult male zebra finch syrinx stained for laminin (Lam, red) to quantify fibre number, cross-sectional area (CSA). Neurofilament staining (NF, green) was used to confirm successful denervation. b, Summed CSA is significantly smaller on the denervated side (Paired two-sided Welch's t-test, p=0.00241, N=4), while c, the number of muscle fibres remains the same (Paired two-sided Welch's t-test, p=0.4385, N=4). ** at p<0.01, n.s. at p≥0.05. Data are presented as mean values ± 1 S.D. Source data are provided as a Source Data file.

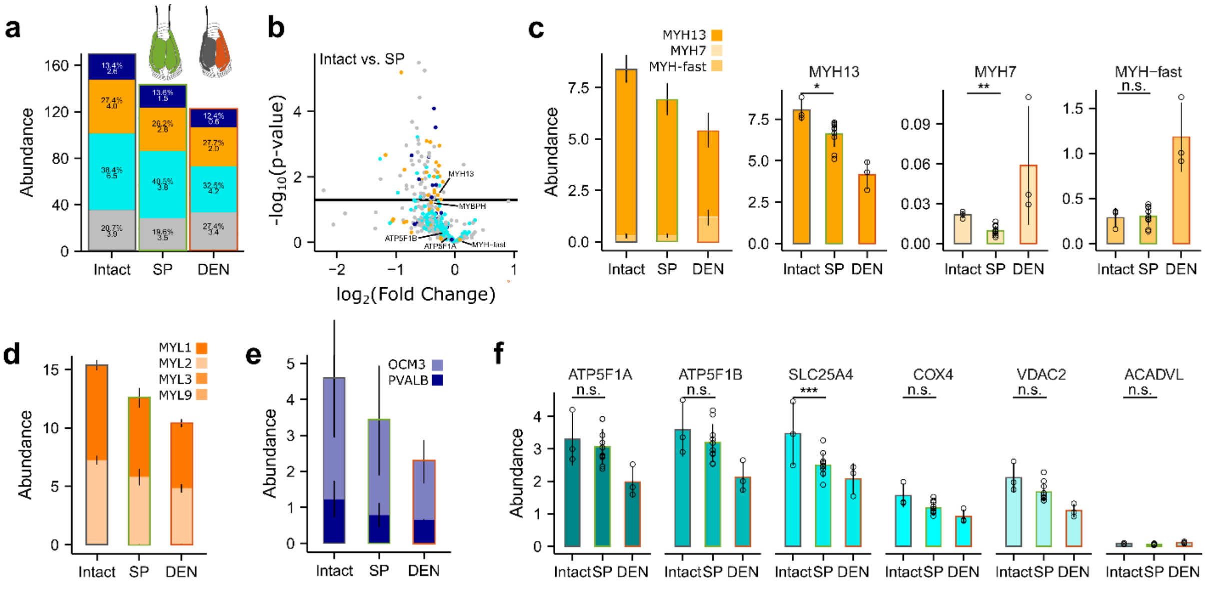

Supplementary Figure 2. Preventing birds from singing for seven days induces changes in protein abundance in the same direction as long-term denervation. a, Singing prevention (green outlines) causes reduction of total protein abundance in comparison to unmanipulated muscles (grey outlines), which is the same direction as observed in long term denervation (orange outlines). Fill colour refers to protein the same protein groups as in **Fig 2**: sarcomeric (orange), mitochondrial (cyan) and calcium handling (blue), other (grey). b, Volcano plot of 450 identified proteins showing statistical significance over magnitude of fold change due to singing prevention. c, The abundance of total MyHC proteins, d, myosin light chains, e, parvalbumins, and f, mitochondrial proteins decreases after denervation. Statistical significance was tested using unpaired, two-sided Welch's t-test between intact (N=3) vocal muscles and vocal muscles from birds that were not allowed to sing for 7 days (N=10) (see **Supplementary Data S1**). * at $p<0.05$, ** at $p<0.01$, *** at $p<0.001$, n.s. at $p \geq 0.05$. The intact and DEN data is replotted from **Fig 2e-k**. Data are presented as mean values ± 1 S.D. Source data are provided as a Source Data file.

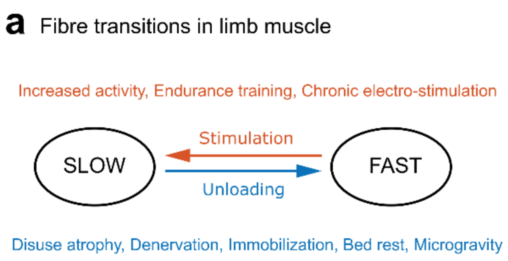

Supplementary Figure 3. Model of neural regulation of vocal muscle fibre types. a, Unloading paradigms, such as disuse, denervation and bed rest in mammalian limb muscles typically transform slow fibre types (Type 1, 2A) into fast fibre types (type 2X, 2B), while reversely training programs typically transform fast into slower fibre types. Based on reference [5]**.** b, Proposed model of fibre transitions in vocal muscle plasticity (larynx and syrinx) due to neural regulation (**Supplementary Table 1**).

3 Supplementary Tables

Supplementary Table 1. Fibre type plasticity in laryngeal and syringeal vocal muscles due to neural modulation. Changes in MyHC composition in loading paradigms gives a mixed image: Chronic electrostimulations shows no detectable shift or a slight increase in slower MyHCs, while cross-innervation with a faster nerve leads to an increase of faster MyHCs, reinnervation leads to slower MyHC composition. In contrary to denervation and unloading studies in general, where neural drive is inevitably decreased, information about the introduced change in neural drive is lacking in loading studies.

**Unloading**	**Larynx**	**Evidence**	**Syrinx**	**Evidence**
CSA	↓	References[6-13]	↓	This study
MyHC isoform/speed	Fast isoform ↓ Slow isoform	References[6,7,13-15] but see[16]	Fast ↓ Slow	This study
Oxidative potential Mitochondria	↓	Reference[9]	↓	This study
**Loading**	**Larynx**		**Syrinx**	
CSA	↑	References[17,18] but see[19]	(↑)	Increase after onset of song learning; Reference[20]
MyHC isoform/speed	↑	Reference[21] but see[17,19]	(↑)	Increased MYH13 expression after onset of song learning; References[1,22]
Oxidative potential Mitochondria	↑	Reference[19]	(↑)	Unusually high mitochondrial VPE and cristae density in freely singing animals; References[1,23]

Supplementary Table 2. Primary antibodies used for immunohistochemistry

Antibody	Target	Gene (human)	Dilution	Clone	Source
**M4276**	Fast MyHC	*MYH1, MYH2*	1:500	MY-32	www.sigmaaldrich.com/DK/en/product/sigma/m4276
**L9393**	Laminin	*LAMA1*	1:500	Polyclonal	www.sigmaaldrich.com/DK/en/product/sigma/l9393
**CBL212**	Neurofilament	*NEFH*	1:500	RT97	www.sigmaaldrich.com/DK/en/product/mm/cbl212

*Official gene nomenclature according to HGNC. (https://www.genenames.org/)

4 Supplementary References

1 Mead, A. F. *et al.* Fundamental constraints in synchronous muscle limit superfast motor control in vertebrates. *Elife* **6**, doi:10.7554/eLife.29425 (2017).

2 Christensen, L. A., Allred, L. M., Goller, F. & Meyers, R. A. Is sexual dimorphism in singing behavior related to syringeal muscle composition? *The Auk* **134**, 710-720, doi:10.1642/auk-17-3.1 (2017).

3 Deshmukh, A. S. *et al.* Deep proteomics of mouse skeletal muscle enables quantitation of protein isoforms, metabolic pathways, and transcription factors. *Mol Cell Proteomics* **14**, 841-853, doi:10.1074/mcp.M114.044222 (2015).

4 Rome, L. C. Design and function of superfast muscles: new insights into the physiology of skeletal muscle. *Annu Rev Physiol* **68**, 193-221, doi:10.1146/annurev.physiol.68.040104.105418 (2006).

5 Ohlendieck, K. Proteomic profiling of skeletal muscle plasticity. *Muscles Ligaments Tendons J* **1**, 119-126 (2011).

6 Caiozzo, V. J., Wu, Y. Z., Baker, M. J. & Crumley, R. Effects of denervation on cell cycle control in laryngeal muscle. *Arch Otolaryngol Head Neck Surg* **130**, 1056-1068, doi:10.1001/archotol.130.9.1056 (2004).

7 Kumai, Y., Ito, T., Matsukawa, A. & Yumoto, E. Effects of denervation on neuromuscular junctions in the thyroarytenoid muscle. *Laryngoscope* **115**, 1869-1872, doi:10.1097/01.mlg.0000177076.33294.89 (2005).

8 Kumai, Y., Ito, T., Udaka, N. & Yumoto, E. Effects of a nerve-muscle pedicle on the denervated rat thyroarytenoid muscle. *Laryngoscope* **116**, 1027-1032, doi:10.1097/01.MLG.0000217588.46645.FA (2006).

9 Li, Z. B., Lehar, M., Samlan, R. & Flint, P. W. Proteomic analysis of rat laryngeal muscle following denervation. *Proteomics* **5**, 4764-4776, doi:10.1002/pmic.200401329 (2005).

10 Miyamaru, S., Kumai, Y., Ito, T. & Yumoto, E. Effects of long-term denervation on the rat thyroarytenoid muscle. *Laryngoscope* **118**, 1318-1323, doi:10.1097/MLG.0b013e31816f693f (2008).

11 Sahgal, V. & Hast, M. H. Effect of denervation on primate laryngeal muscles: a morphologic and morphometric study. *J Laryngol Otol* **100**, 553-560, doi:10.1017/s0022215100099667 (1986).

12 Shindo, M. L., Herzon, G. D., Hanson, D. G., Cain, D. J. & Sahgal, V. Effects of denervation on laryngeal muscles: a canine model. *Laryngoscope* **102**, 663-669, doi:10.1288/00005537-199206000-00012 (1992).

13 Wu, Y. Z., Baker, M. J., Marie, J. P., Crumley, R. & Caiozzo, V. J. The plasticity of denervated and reinnervated laryngeal muscle: focus on single-fiber myosin heavy-chain isoform expression. *Arch Otolaryngol Head Neck Surg* **130**, 1070-1082, doi:10.1001/archotol.130.9.1070 (2004).

14 Bijangi-Vishehsaraei, K., Blum, K., Zhang, H., Safa, A. R. & Halum, S. L. Microarray Analysis Gene Expression Profiles in Laryngeal Muscle After Recurrent Laryngeal Nerve Injury. *Ann Otol Rhinol Laryngol* **125**, 247-256, doi:10.1177/0003489415608866 (2016).

15 Shiotani, A. & Flint, P. W. Myosin heavy chain composition in rat laryngeal muscles after denervation. *Laryngoscope* **108**, 1225-1229 (1998).

16 DelGaudio, J. M. & Sciote, J. J. Changes in myosin expression in denervated laryngeal muscle. *Ann Otol Rhinol Laryngol* **106**, 1076-1081, doi:10.1177/000348949710601212 (1997).

17 Karbiener, M. *et al.* Reversing Age Related Changes of the Laryngeal Muscles by Chronic Electrostimulation of the Recurrent Laryngeal Nerve. *PLoS One* **11**, e0167367, doi:10.1371/journal.pone.0167367 (2016).

18 Stemple, J. *et al.* Response of aging laryngeal muscles to chronic electrical stimulation. *The FASEB Journal* **29**, 815.811 (2015).

19 McMullen, C. A. *et al.* Chronic stimulation-induced changes in the rodent thyroarytenoid muscle. *J Speech Lang Hear Res* **54**, 845-853, doi:10.1044/1092-4388(2010/10-0127) (2011).

20 Godsave, S. F., Lohmann, R., Vloet, R. P. & Gahr, M. Androgen receptors in the embryonic zebra finch hindbrain suggest a function for maternal androgens in perihatching survival. *J Comp Neurol* **453**, 57-70, doi:10.1002/cne.10391 (2002).

21 Paniello, R. C., West, S. E. & Lee, P. Laryngeal reinnervation with the hypoglossal nerve. I. Physiology, histochemistry, electromyography, and retrograde labeling in a canine model. *Ann Otol Rhinol Laryngol* **110**, 532-542, doi:10.1177/000348940111000607 (2001).

22 Adam, I. & Elemans, C. P. H. Increasing Muscle Speed Drives Changes in the Neuromuscular Transform of Motor Commands during Postnatal Development in Songbirds. *J Neurosci* **40**, 6722-6731, doi:10.1523/JNEUROSCI.0111-20.2020 (2020).

23 Nielsen, J. *et al.* Plasticity in mitochondrial cristae density allows metabolic capacity modulation in human skeletal muscle. *J Physiol* **595**, 2839-2847, doi:10.1113/JP273040 (2017).
