# Supplementary material for: Daily vocal exercise is necessary for peak performance singing in a songbird
Source: Nat Commun. 2023 Dec 12;14:7787. doi: 10.1038/s41467-023-43592-6 (PMC10716414; doi:10.1038/s41467-023-43592-6)
Supplement: Supplementary file 3 — Description of Additional Supplementary Files [file 41467_2023_43592_MOESM3_ESM.pdf]

Description of Additional Supplementary Files

File Name: Supplementary Data 1
Description: Summary of statistical tests.

File Name: Supplementary Data 2
Description: Proteomics data: LC peaks of intact and denervated DTB muscle. LC peaks of DTB muscle after 7 days of singing prevention.
