# Supplementary material for: Daily vocal exercise is necessary for peak performance singing in a songbird
Source: Nat Commun. 2023 Dec 12;14:7787. doi: 10.1038/s41467-023-43592-6 (PMC10716414; doi:10.1038/s41467-023-43592-6)
Supplement: Supplementary file 6 — Reporting Summary [file 41467_2023_43592_MOESM6_ESM.pdf]

nature portfolio

Corresponding author(s): Iris Adam
Coen P.H. Elemans

Last updated by author(s): Oct 31, 2023

Reporting Summary

Nature Portfolio wishes to improve the reproducibility of the work that we publish. This form provides structure for consistency and transparency in reporting. For further information on Nature Portfolio policies, see our Editorial Policies and the Editorial Policy Checklist.

Statistics

For all statistical analyses, confirm that the following items are present in the figure legend, table legend, main text, or Methods section.

n/a	Confirmed	
☐	☒	The exact sample size (*n*) for each experimental group/condition, given as a discrete number and unit of measurement
☐	☒	A statement on whether measurements were taken from distinct samples or whether the same sample was measured repeatedly
☐	☒	The statistical test(s) used AND whether they are one- or two-sided<br>*Only common tests should be described solely by name; describe more complex techniques in the Methods section.*
☐	☒	A description of all covariates tested
☐	☒	A description of any assumptions or corrections, such as tests of normality and adjustment for multiple comparisons
☐	☒	A full description of the statistical parameters including central tendency (e.g. means) or other basic estimates (e.g. regression coefficient) AND variation (e.g. standard deviation) or associated estimates of uncertainty (e.g. confidence intervals)
☐	☒	For null hypothesis testing, the test statistic (e.g. *F*, *t*, *r*) with confidence intervals, effect sizes, degrees of freedom and *P* value noted<br>*Give P values as exact values whenever suitable.*
☒	☐	For Bayesian analysis, information on the choice of priors and Markov chain Monte Carlo settings
☒	☐	For hierarchical and complex designs, identification of the appropriate level for tests and full reporting of outcomes
☒	☐	Estimates of effect sizes (e.g. Cohen's *d*, Pearson's *r*), indicating how they were calculated

Our web collection on statistics for biologists contains articles on many of the points above.

Software and code

Policy information about availability of computer code

Data collection	Muscle physiology: Custom written Matlab code<br>Sound recording: Sound Analysis Pro 2011 Recorder version 1.085<br>Image acquisition: Leica LASX, 2018, 3.6.0.<br>Proteomics: peptides were separated by ultra-high pressure liquid chromatography and directly infused into a Q Exactive Hybrid Quadrupole-Orbitrap Mass Spectrometer (Thermo Fisher Scientific). Data were collected in data dependent mode and recorded in .raw files.<br>Female preference tests: Custom written software log keypecking events, trigger stimulus playback and switch stimulus-key assignment on a daily basis.
Data analysis	Analysis of proteomics data: Peptides were identified and liquid chromatography (LC) peak areas were determined using Proteome Discoverer 2.2 to search against the zebra finch (Taeniopygia guttata) database downloaded from UniProt (11/22/2022)<br>All other data analysis and processing was performed using custom written Matlab (Version 2022b, MathWorks, Inc.) or R (4.2.2) code.<br>Fundamental frequency of song syllables was detected using the Matlab implementation of the YIN algorithm.

For manuscripts utilizing custom algorithms or software that are central to the research but not yet described in published literature, software must be made available to editors and reviewers. We strongly encourage code deposition in a community repository (e.g. GitHub). See the Nature Portfolio guidelines for submitting code & software for further information.

Data

Policy information about availability of data

All manuscripts must include a data availability statement. This statement should provide the following information, where applicable:

- Accession codes, unique identifiers, or web links for publicly available datasets
- A description of any restrictions on data availability
- For clinical datasets or third party data, please ensure that the statement adheres to our policy

The proteomic data generated in this study have been deposited in the MassIVE database (Dataset: MSV000091352) (https://massive.ucsd.edu/ProteoSAFe/private-dataset.jsp?task=1a7f2f007a3247bf998adde62872cd2e). The processed proteomics data are available as Supplementary Data 2. Detailed statistical results are provided in Supplementary Data 1. Source data are provided as a Source Data file. All other data are available from the corresponding authors upon reasonable request.

Human research participants

Policy information about studies involving human research participants and Sex and Gender in Research.

Reporting on sex and gender	N/A
Population characteristics	N/A
Recruitment	N/A
Ethics oversight	N/A

Note that full information on the approval of the study protocol must also be provided in the manuscript.

Field-specific reporting

Please select the one below that is the best fit for your research. If you are not sure, read the appropriate sections before making your selection.

[x] Life sciences [ ] Behavioural & social sciences [ ] Ecological, evolutionary & environmental sciences

For a reference copy of the document with all sections, see nature.com/documents/nr-reporting-summary-flat.pdf

Life sciences study design

All studies must disclose on these points even when the disclosure is negative.

Sample size	No sample sizes were determined. We used samples sizes in the range of those used in previous studies conducting similar analyses (Adam & Elemans 2020, Honarmand et al 2015).
Data exclusions	Syllables with spurious or non-robust pitch detections were excluded from the data.
Replication	All results were collected independently from multiple animals. The number of repetitions corresponds to the number of animals (N). The reported effects were found in all animals.
Randomization	Initial key, stimulus assignment in the female preference test was randomized. All custom analysis scripts were automated and thus no blinding was required. Animals were assigned randomly to experimental groups.
Blinding	For the female preference tests experimenters were blind to the stimulus-origin (pre singing prevention/post singing prevention) during training, testing and analysis. Experimenters were blind to sample identity during processing of tissue samples, data collection and analysis of proteomics data.

Reporting for specific materials, systems and methods

We require information from authors about some types of materials, experimental systems and methods used in many studies. Here, indicate whether each material, system or method listed is relevant to your study. If you are not sure if a list item applies to your research, read the appropriate section before selecting a response.

Materials & experimental systems

n/a	Involved in the study	
	x	Antibodies
x		Eukaryotic cell lines
x		Palaeontology and archaeology
	x	Animals and other organisms
x		Clinical data
x		Dual use research of concern

Methods

n/a	Involved in the study	
x		ChIP-seq
x		Flow cytometry
x		MRI-based neuroimaging

Antibodies

Antibodies used	M4276, MY-32, www.sigmaaldrich.com/DK/en/product/sigma/m4276, LOT number: 0000120824, dilution: (1:500)<br>L9393 anti-Laminin, www.sigmaaldrich.com/DK/en/product/sigma/l9393, LOT number: 099M4886V, dilution: (1:500)<br>CBL212 anti-Neurofilament, www.sigmaaldrich.com/DK/en/product/mm/cbl212, LOT number: 3059117, dilution: (1:500)<br>Goat anti-Mouse IgG Alexa Fluor 488, (A-11029, Invitrogen by Thermo Fisher Scientific, Rockford, USA), LOT number: 2066709, dilution: (1:1000)<br>Goat anti-Rabbit IgG Alexa Fluor 568 (A-11036, Invitrogen by Thermo Fisher Scientific, Rockford, USA), LOT number: 2045347, dilution: (1:1000)
Validation	All antibodies have been validated and used previously (10.1016/j.cub.2021.05.008, 10.7554/eLife.29425).

Animals and other research organisms

Policy information about studies involving animals; ARRIVE guidelines recommended for reporting animal research, and Sex and Gender in Research

Laboratory animals	Zebra finches (Taeniopygia castanotis), adult (>100 days post hatching) females (N=13) and males 25 days post hatching (N=8, juveniles) and >100 days post hatching (N=71, adults).
Wild animals	The study did not involve wild animals
Reporting on sex	All data except the experiment to test for female preference were conducted on male animals as in this species, only males display the behavior we investigated (learned song). Sex was determined phenotypically (plumage is sexually dimorphic).
Field-collected samples	The study did not involve samples collected from the field.
Ethics oversight	All experiments and procedures were performed in accordance with the Danish Animal Experiments Inspectorate Copenhagen, Denmark (2019-15-0201-00308). Female preference tests were approved by the committee for animal experimentation at Leiden University and the Centrale Commissie Dierproeven (CCD) of the Netherlands (1160020186606) and monitored by the Animal Welfare Body of Leiden University, in accordance with national and European legislation.

Note that full information on the approval of the study protocol must also be provided in the manuscript.
